# Online Patient Education Materials Related to Lipoprotein(a): Readability Assessment

**DOI:** 10.2196/31284

**Published:** 2022-01-11

**Authors:** Keon Pearson, Summer Ngo, Eson Ekpo, Ashish Sarraju, Grayson Baird, Joshua Knowles, Fatima Rodriguez

**Affiliations:** 1 Department of Medicine Stanford University Stanford, CA United States; 2 Division of Cardiovascular Medicine and the Cardiovascular Institute Stanford University Stanford, CA United States; 3 Rhode Island Hospital and Diagnostic Imaging Warren Alpert Medical School Brown University Providence, RI United States

**Keywords:** lipoprotein(a), readability, online patient education material, health education, health literacy

## Abstract

**Background:**

Lipoprotein(a) (Lp(a)) is a highly proatherogenic lipid fraction that is a clinically significant risk modifier. Patients wanting to learn more about Lp(a) are likely to use online patient educational materials (OPEMs). However, the readability of OPEMs may exceed the health literacy of the public.

**Objective:**

This study aims to assess the readability of OPEMs related to Lp(a). We hypothesized that the readability of these online materials would exceed the sixth grade level recommended by the American Medical Association.

**Methods:**

Using an online search engine, we queried the top 20 search results from 10 commonly used Lp(a)-related search terms to identify a total of 200 websites. We excluded duplicate websites, advertised results, research journal articles, or non–patient-directed materials, such as those intended only for health professionals or researchers. Grade level readability was calculated using 5 standard readability metrics (automated readability index, SMOG index, Coleman-Liau index, Gunning Fog score, Flesch-Kincaid score) to produce robust point (mean) and interval (CI) estimates of readability. Generalized estimating equations were used to model grade level readability by each search term, with the 5 readability scores nested within each OPEM.

**Results:**

A total of 27 unique websites were identified for analysis. The average readability score for the aggregated results was a 12.2 (95% CI 10.9798-13.3978) grade level. OPEMs were grouped into 6 categories by primary source: industry, lay press, research foundation and nonprofit organizations, university or government, clinic, and other. The most readable category was OPEMs published by universities or government agencies (9.0, 95% CI 6.8-11.3). The least readable OPEMs on average were the ones published by the lay press (13.0, 95% CI 11.2-14.8). All categories exceeded the sixth grade reading level recommended by the American Medical Association.

**Conclusions:**

Lack of access to readable OPEMs may disproportionately affect patients with low health literacy. Ensuring that online content is understandable by broad audiences is a necessary component of increasing the impact of novel therapeutics and recommendations regarding Lp(a).

## Introduction

Lipoprotein(a) (Lp(a)) is a highly proatherogenic lipid fraction that is increasingly recognized as a clinically significant risk modifier. The 2018 American College of Cardiology and the American Heart Association guidelines recommend using Lp(a) as a risk-enhancing factor favoring initiation of statin therapy in persons with intermediate atherosclerotic cardiovascular disease (ASCVD) risk [1]. This recommendation is based on data in recent years showing a strong genetic determination of Lp(a) levels; limited modifiability with diet, exercise, and medication; and an increased risk of ASCVD independent of traditional risk factors [2]. National cohort studies using Mendelian randomization estimate that reducing Lp(a) by 50 mg/dl and 99 mg/dl could reduce major adverse cardiac events by 20% and 40%, respectively [3]. Statins do not lower Lp(a), and there are currently no Food and Drug Administration–approved therapies to lower Lp(a) specifically. PCSK-9 inhibitors result in a modest reduction in Lp(a) and may be associated with a reduction in major adverse cardiovascular events for patients with elevated baseline Lp(a) who have experienced acute coronary syndrome [4,5]. Antisense oligonucleotides targeting expression of Lp(a) are in phase III clinical trials [6,7]. The National Lipid Association published guidance in 2019 for clinicians using Lp(a) in clinical practice, and as novel therapeutics are approved, interest in this biomarker is expected to increase in the coming years [8]. Patients may be especially interested in learning their Lp(a) levels after encountering stories of public figures experiencing early ASCVD in the setting of an elevated Lp(a) [9].

Patients wanting to learn more about Lp(a) are likely to use online patient educational materials (OPEMs) [10]. However, patient health literacy may influence utilization of evidence-based OPEMs [11,12]. The American Medical Association (AMA) recommends writing health information for patients at the sixth grade level or below to ensure broad comprehension [13]. Although OPEMs influence patient decision-making, the readability of OPEMs generally exceeds the health literacy of the public [14-16]. Patients with lower health literacy have been noted to have poorer overall health and higher mortality, and this is notably true as well among patients from racial and ethnic minority backgrounds, partially explaining the racial disparities in some outcomes [17]. The disparate impact of elevated Lp(a) in racial/ethnic groups and the lack of standardization of the use of this biomarker in clinical practice make it even more pressing to ensure that evidence-based materials are appropriately written for the public. Thus, we sought to quantify the readability of frequently accessed OPEMs about Lp(a).

## Methods

### Data Acquisition and Refinement

We used Google, the largest online search engine, to query the first 20 results for each of the following 10 search terms: “lipoprotein(a),” “lipoprotein(a) cardiovascular risk,” “lipoprotein(a) elevated,” “lipoprotein(a) high,” “lipoprotein(a) levels,” “lipoprotein(a) screening,” “lipoprotein(a) test,” “Lp little a,” “Lp(a),” and “Lp(a) screening” [18]. Location, cookies, and user account information were disabled beforehand to avoid search bias. All 200 websites were accessed and downloaded as PDFs on November 5 to 6, 2020. OPEMs were defined as materials intended for patients and the public. Two independent reviewers reviewed each source’s mission statement or informational page to determine whether it was patient-facing. Research journal articles, advertised results, results failing to contain material on Lp(a), and non–patient-directed sources such as those intended only for health professionals or researchers were excluded.

This research is exempt from human participant institutional review board approval as no human participant data were used, and only publicly available OPEMs were included in the analysis.

OPEMs were grouped into 6 categories by primary source. *Industry OPEMs* were published by for-profit companies or offered a proprietary test or service related to Lp(a). *Lay press OPEMs* were published by news organizations and health care reporters that do not have a specific research or scientific focus. *Research foundations and nonprofit OPEMs* were published by health-related organizations that have a specific research or scientific focus. *University or government OPEMs* were published by academic institutions or national, state, or local government agencies. *Clinic OPEMs* were published by Williams Integracare clinic, an independently owned family medicine, chiropractic, and physical therapy clinic in St. Cloud, MN. *Other* includes Wikipedia.org, a crowdsourced online free encyclopedia, which published an article on Lp(a).

### Readability Assessment

Websites meeting the criteria for OPEMs were converted into plain text in separate Word (Microsoft Corporation) documents and prepared in accordance with recommendations from the Centers for Medicare and Medicaid Services prior to scoring readability [19]. Using methods consistent with prior readability assessment studies we removed advertisements, videos, images, figures, captions, hyperlinks, disclaimers, copyright notices, acknowledgments, and citations. Periods were used to denote the end of all sentences; all other punctuation were removed. Symbols and numerals were spelled out to avoid artificial increases in reading level. Readability was assessed with Readable.com, as done in prior literature [16].

### Statistical Methods

Similar to prior readability studies, grade-level readability was calculated using 5 standard readability metrics (automated readability index, SMOG index, Coleman-Liau index, Gunning Fog score, Flesch-Kincaid score) to produce robust point (mean) and interval (CI) estimates of readability. Averaging across multiple readability metrics has been demonstrated to yield more reliable results than relying on a single readability metric [20]. This was done to minimize bias because no single readability metric has been established as a “gold standard”; each readability metric is calculated differently and varies in limitations [21]. Generalized estimating equations were used to model grade-level readability by each search term, with the 5 readability scores nested within each OPEM [16]. Readability analyses were conducted with SAS Software 9.4 (SAS Institute), with sandwich estimation and the GLIMMIX procedure. All interval estimates were calculated for 95% confidence. The interval estimates reflect the variability of readability for the 5 readability metrics for each OPEM.

## Results

**Figure 1:**
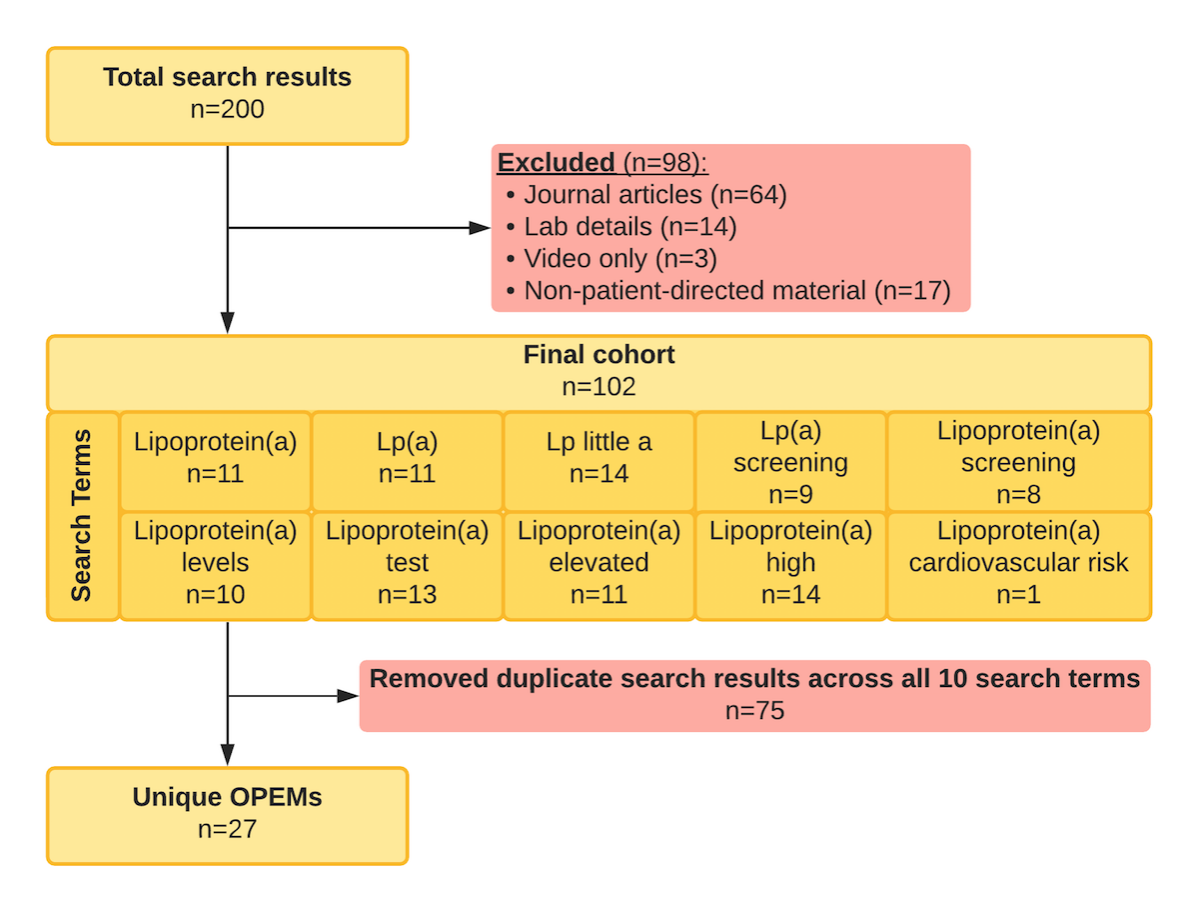
Material selection and exclusion for OPEMs related to lipoprotein(a). OPEM: online patient education material.

Figure 1 summarizes the selection Lp(a) OPEMs for analysis. From an initial sample of 200 total search results across all 10 search terms, 102 results met inclusion criteria. A total of 75 duplicate results were then removed. There were 27 unique OPEMs included in the readability analysis.

Among the 27 OPEMs, the largest category of were university and government sources, which included 30% (n=8) of unique search results. The next largest categories were research and nonprofit foundations and industry publications, which each comprised 22% (n=6) of the results. The third largest category of OPEMs were articles in the lay press, which comprised 19% (n=5/27) of results. The smallest categories were clinic publications and other, both of which had 1 (3%) website (Williams Integracare Clinic and Wikipedia, respectively).

**Figure 2:**
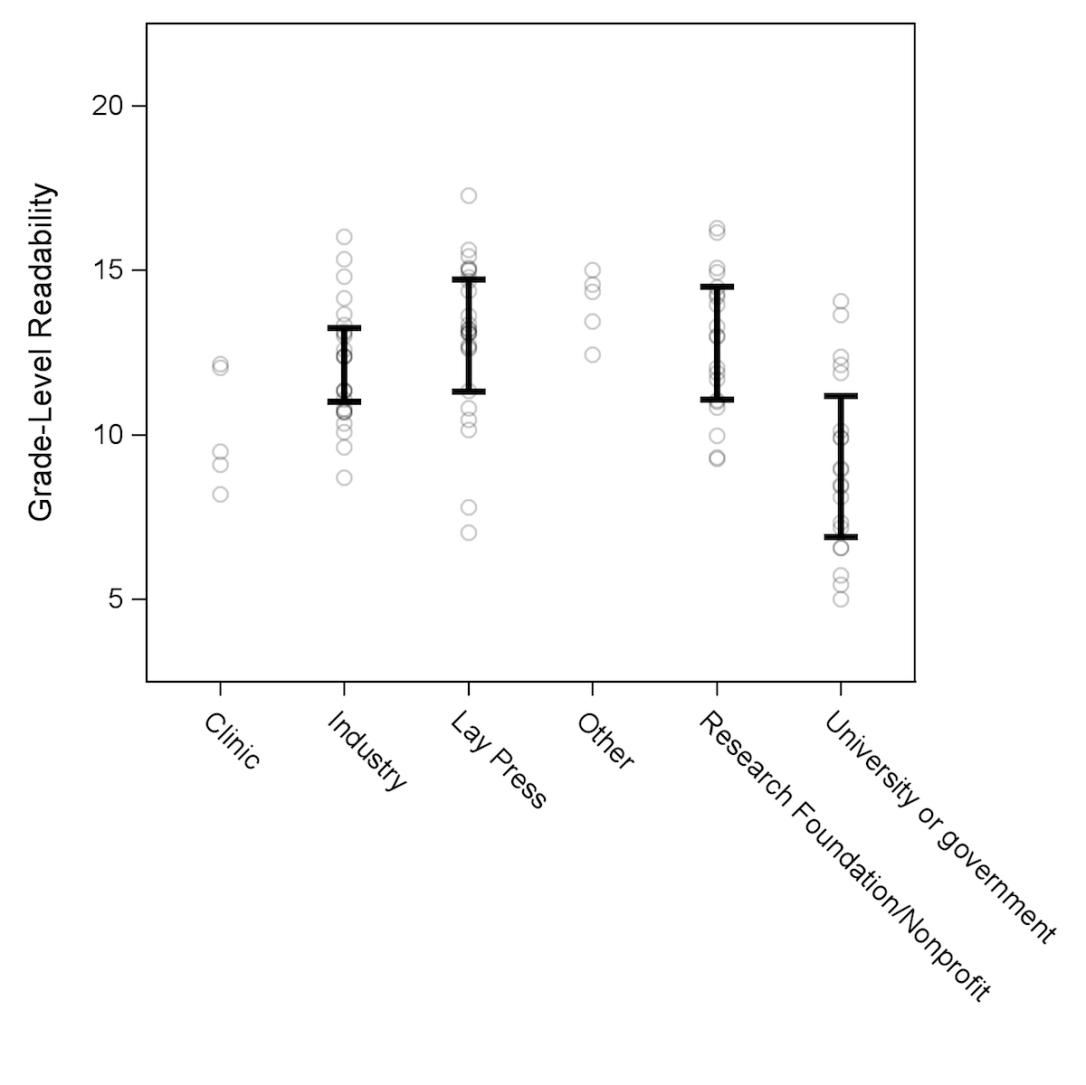
Average grade level readability of online patient education material (OPEM) by category of publication. Each circle represents a readability score for one OPEM, with a total of five readability scores for each unique OPEM. 95% CIs are included for all categories except “Clinic” and “Other,” which only had one unique OPEM each. University and government sources were significantly more readable than research and nonprofit foundation, industry, and lay press sources.

The average readability score across unique websites was at a 12.2 (95% CI 10.9798-13.3978) grade level. The most readable category was OPEMs published by universities or government agencies (9.0, 95% CI 6.8-11.3). The least readable OPEMs on average were the ones published by lay press (13.0, 95% CI 11.2-14.8). Research and nonprofit foundations (12.8, 95% CI 11.0-14.6) and industry (12.1, 95% CI 10.9-13.3) had intermediate readability. Clinic (10.2) and other publications (14) had 1 site per category and therefore are not averaged figures. Figure 2 summarizes the readability scores by category of OPEMs.

**Figure 3:**
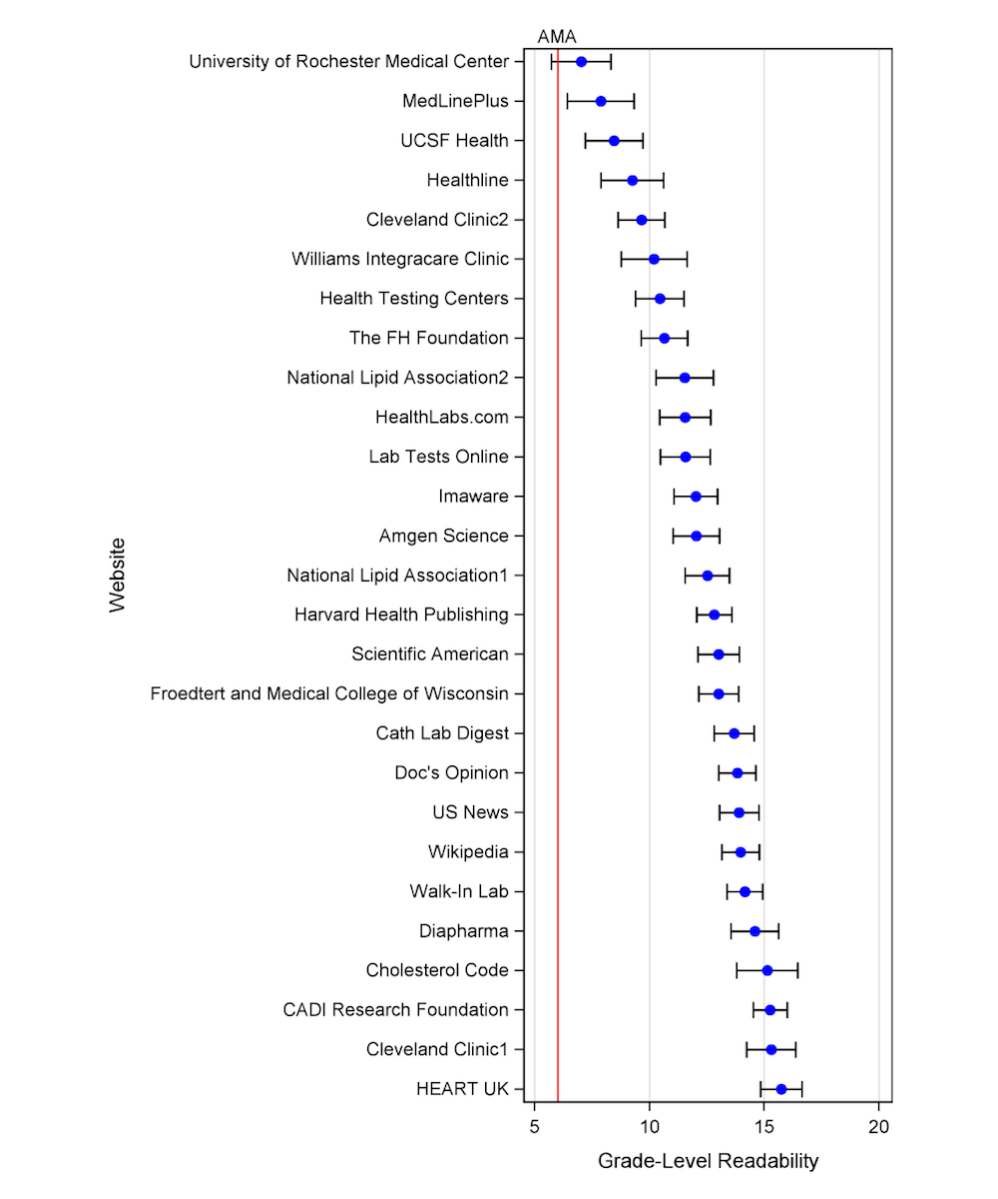
Readability rankings by search result. Each blue dot corresponds to the mean readability based on the average of five standard readability scores (automated readability index, SMOG index, Coleman-Liau index, Gunning fog score, Flesch-Kincaid score) with whiskers representing the range of readability scores. The red vertical line corresponds to the AMA sixth grade level readability target. Cleveland Clinic1: Elevated Lipoprotein(a): Is a Long-Sought Treatment Finally on the Way?; Cleveland Clinic2: Why Would My Doctor Order a Lipoprotein(a) Blood Test?; National Lipid Association1: lipoprotein(a) Screening for Individuals at High ASCVD Risk; National Lipid Association2: Elevated Lipoprotein (a) Patient-Centered Education From the National Lipid Association. AMA: American Medical Association.

Of the 27 unique search results, only 1 site, the University of Rochester Medical Center (7.0, 95% CI 5.7-8.3), had the sixth grade level recommended by the AMA within its 95% CI. The least readable OPEM was the HEART UK’s Lp(a) general information site (15.7, 95% CI 14.8-16.6). The readability of each unique website is shown in Figure 3.

Table 1 highlights excerpts from Lp(a) OPEMs across readability levels.

**Table 1:** Search concepts and excerpts of readable and nonreadable quotes from OPEM.

Concept	Excerpt from less readable OPEM^a^	Excerpt from more readable OPEM
Lp(a)^b^ contains both a lipid and a protein carrier	“Lipoprotein(a), or Lp(a), is a distinctive particle with two components: a lipoprotein core that resembles LDL, along with a shell that contains apolipoprotein(a), or apo(a).” [22]	“Lipoproteins are substances made up of protein and fat” [23]
Lp(a) is a proatherogenic lipoprotein fraction	“High levels of LP(a) have now been identified as an independent risk factor in cardiovascular disease, with a causal link to atherosclerosis (furring up of arteries), heart attacks, strokes, aortic valve disease and heart failure.” [24]	“High levels of Lp(a) can create plaque in your blood vessels.” [25]
High-risk populations should be screened for Lp(a)	“Measurement of lipoprotein(a) is now recommended in several patient subgroups… patients with premature atherosclerosis; patients with a strong family history of premature coronary heart disease (CHD); patients with elevated LDL-C and greater than or equal to two risk factors; patients who have had coronary angioplasty in whom lipoprotein(a) excess may increase the risk of restenosis; patients who have undergone coronary bypass graft surgery in whom Lp(a) excess may be associated with graft stenosis.” [26]	“You may need this test if you have: Heart disease, despite normal results on other lipid tests, High cholesterol, despite maintaining a healthy diet A family history of heart disease, especially heart disease that has occurred at an early age and/or sudden deaths from heart disease.” [27]
There is no widely implemented standard for measuring Lp(a)	“Although the reference material for the accurate measurement of Lp(a) … has been available for many years, many commercial laboratories have not changed their reagents and testing methods and continue to use old reagents and methods resulting in inaccurate results. Accordingly, results of Lp(a) measurements by different labs are not comparable and some of them are clearly inaccurate.” [28]	“Note: Normal value ranges may vary slightly among different laboratories. Talk to your doctor about the meaning of your specific test results.” [29]
There are no drugs demonstrated to improve outcomes in patients with elevated Lp(a)	“Results using statin medications have been mixed in most trials … In severe cases, such as familial hypercholesterolemia or treatment- resistant hypercholesterolemia, lipid apheresis may dramatically reduce Lp(a) … Other medications that are in various stages of development include thyromimetics, cholesterol-ester-transfer protein (CETP inhibitors), anti-sense oligonucleopeptides, and proprotein convertase subtilisin/kexin type 9 (PCSK-9) inhibitors.” [30]	“Medications/treatments in current use that lower Lp(a) also lower cholesterol. There are apheresis and niacin. These both have substantial side effects. PCSK9 inhibitors lower Lp(a) while lowering LDL cholesterol. Statins have no effect on Lp(a).” [31]

^a^OPEM: online patient educational material.

^b^Lp(a): lipoprotein(a).

A list of the OPEM sources used for this study can be found in Multimedia Appendix 1.

## Discussion

### Principal Results

We found that the average reading grade level of OPEMs pertaining to Lp(a) generally exceeded AMA readability recommendations that OPEMs be written at or below a sixth grade reading level to be accessible to the public. Average grade-level readability of OPEMs on Lp(a) was a 12.2 (95% CI 10.9798-13.3978) grade level, exceeding the average reading level of US adults (eighth grade) [13]. Of 27 unique websites reviewed, only 1 website had a lower bound of reading grade level (5.7) that was below the sixth grade reading level recommendation. Our results suggest that the overwhelming majority of Lp(a) OPEMs are written at a reading level that is too high for the minimally health literate members of the public.

These findings have several important implications for how patients may make decisions about this important, proatherogenic lipid fraction that is receiving increased attention. Patients frequently use the internet to supplement health information from their clinicians [32]. If presented clearly, online health information can be a valuable patient resource. Patients who feel more informed are more comfortable asking their provider questions and report better understanding of their providers’ explanations and greater self-confidence in making health care decisions [33]. A 2018 cross-sectional survey found that patients who searched online for health information to solve their medical problems were also significantly more likely to change their medical decision based on information gathered [34]. It is noteworthy that OPEMs in the academic and government categories were the most readable. Academic and government sites are regarded as reliable sources given that many organizations seek to advance public health and knowledge [35]. Given the major influence of online health information on decision-making, these academic and government websites may also benefit from direct guidance to craft OPEMs with readability targets in view. Despite the importance of these OPEMs in reaching the lay community, research foundations and nonprofit sites demonstrate the largest gaps between patient reading skills and OPEMs reading level on Lp(a).

The less readable OPEMs tend to cover topics in greater depth, including nuances around Lp(a) measurement and standardization, and the role of Lp(a) in thrombogenesis and wound healing. Thus, there appears to be a trade-off between readability and comprehensiveness in OPEMs.

These findings align with results from prior studies across a broad range of health conditions that show OPEMs commonly exceed the recommended readability level. Ayyaswami et al [36] showed that greater than 99% of OPEMs relating to cardiovascular disease were written above the grade level recommended by the AMA [35]. OPEMs are frequently written at a reading level too difficult for the public to comprehend, and low readability levels have been documented across disciplines including common topics related to surgery, oncology, and radiology [37,38].

The percentage of adults with below basic health literacy is considerably higher for populations who identify as Black (24%), Hispanic (41%), American Indian/Alaska Native (25%), and Asian/Pacific Islander (13%) compared to non-Hispanic White (9%) [39]. Similarly, over half of adults older than 65 years were found to have less than a basic health literacy level [39]. These are the very populations known to face a disproportionate burden of cardiovascular risk. Our current risk prediction models such as the Pooled Cohort Equations do not adequately capture the risk to heterogenous racial/ethnic groups [1,40-42]. The importance of reaching these populations is further increased given that Lp(a) is known to circulate at higher levels in patients of African and South Asian descent [43,44].

Many clinical preventive and screening services are underused by historically marginalized racial/ethnic communities and older adults due to inadequate health care access and low health literacy, but we suggest that providing more readable OPEMs may help bridge this gap in care. In the interim, our study findings remind clinicians to consider the readability of OPEMs and patient literacy when recommending Lp(a) evaluation. Shared decision-making requires adequate understanding of the risks and benefits of any diagnostic testing or risk stratification procedure. Actionability of OPEMs on Lp(a) is presently limited by the lack of approved therapies; however, with changes in emerging therapies and practice guidelines, understandability and actionability will be important parameters to assess systematically using the Patient Education Materials Assessment Tool in future studies.

A strength of our study is that we incorporated readability results from five different standard readability metrics, which allows us to have a robust evaluation of readability regardless of the number of websites evaluated. All of the reading grade level estimates supported our hypothesis that Lp(a) OPEMs are written above the recommended sixth grade reading level. We also included a thorough review of possible patient queries by analyzing 200 search results for 10 commonly used search terms. Finally, we included examples of communication of a concept from both more readable and less readable OPEMs. This may serve as a real-world, practical guide for creators of OPEMs seeking to choose words and phrases that will be accessible to a broad audience.

### Limitations

Our study should be interpreted in the context of certain limitations. We did not account for other search engines besides Google. This limitation is somewhat mitigated by the fact that 88% of global internet users use Google as their most frequent search engine [18]. As with other OPEM readability studies, the readability metrics used here do not consider the inherent complexity of some medical terms. Polysyllabic words and longer words are automatically rated as more complex and less understandable than short or monosyllabic words, which in medicine does not always hold true.

### Conclusions

In conclusion, we found that the grade level readability of OPEMs relating to Lp(a) generally substantially exceeded the sixth grade reading level recommended by the AMA. This gap in readability may disproportionately affect patients with low health literacy. Creators of OPEMs should be mindful of the readability of their content. Ensuring that online content is understandable by broad audiences is a necessary component of increasing the impact of novel therapeutics and recommendations regarding Lp(a).

## Appendix

Multimedia Appendix 1 Included online patient educational materials URLs.

## Abbreviations

AMA: American Medical Association
ASCVD: atherosclerotic cardiovascular disease
Lp(a): lipoprotein(a)
OPEM: online patient educational material
