# Supplementary material for: Online Patient Education Materials Related to Lipoprotein(a): Readability Assessment
Source: J Med Internet Res. 2022 Jan 11;24(1):e31284. doi: 10.2196/31284 (PMC8790699; doi:10.2196/31284)
Supplement: Multimedia Appendix 1 [file jmir_v24i1e31284_app1.docx]

| Source | Website | Category |
| --- | --- | --- |
| CADI Research Foundation | <https://cadiresearch.org/topic/lipoproteina/lpa-testing-2> | Research Foundation/Non-profit |
| Diapharma | <https://diapharma.com/lipoprotein-a/> | Industry |
| Williams Integracare Clinic | https://integracareclinics.com/lipoprotein-little-a-lpa/ | Clinic |
| The FH Foundation | https://thefhfoundation.org/lipoprotein-a-5-things | Research Foundation/Non-profit |
| Scientific American | https://blogs.scientificamerican.com/observations/lipoprotein-little-a-can-cause-more-than-a-little-damage-to-the-heart/ | Lay Press |
| Cholesterol Code | https://cholesterolcode.com/the-big-deal-about-lipoproteina/ | Lay Press |
| Cath Lab Digest | https://www.cathlabdigest.com/content/little-known-cvd-risk-factor-target-potential-new-treatment | Research Foundation/Non-profit |
| HealthLabs. com | https://www.healthlabs.com/lipoprotein-a-testing-lpa | Industry |
| Health Testing Centers | https://www.healthtestingcenters.com/test/lipoproteina/ | Industry |
| Walk-in Lab | https://www.walkinlab.com/products/view/lipoprotein-a-blood-test | Industry |
| Imaware | https://www.imaware.health/at-home-blood-test/cardiovascular-lipoproteina-test | Industry |
| US News | https://health.usnews.com/health-care/for-better/articles/2018-08-10/lipoprotein-a-the-other-bad-cholesterol | Lay Press |
| National Lipid Association | <https://www.lipid.org/sites/default/files/elevated_lipoprotein_a.pdf> | Research Foundation/Non-profit |
| Harvard Health Publishing | https://www.health.harvard.edu/heart-health/a-possible-culprit-in-early-heart-attacks | University or government |
| Wikipedia | https://en.wikipedia.org/wiki/Lipoprotein(a) | Other |
| University of Rochester Medical Center | https://www.urmc.rochester.edu/encyclopedia/content.aspx?contenttypeid=167&contentid=lpa_cholesterol | University or government |
| UCSF Health | https://www.ucsfhealth.org/medical-tests/007262 | University or government |
| MedLinePlus | https://medlineplus.gov/lab-tests/lipoprotein-a-blood-test/ | University or government |
| Lab Tests Online | https://labtestsonline.org/tests/lipoprotein-a | Research Foundation/Non-profit |
| Healthline | https://www.healthline.com/health/lipoprotein-a | Lay Press |
| Heart UK | https://www.heartuk.org.uk/genetic-conditions/high-lipoproteina | University or government |
| Froedtert Medical College of Wisconsin | https://www.froedtert.com/stories/lipoprotein-a-other-bad-cholesterol-predict-heart-disease-risk | University or government |
| Doc's Opinion | https://www.docsopinion.com/health-and-nutrition/lipids/lipoprotein-a/ | Lay Press |
| Cleveland Clinic “Why order test” | <https://health.clevelandclinic.org/why-would-my-doctor-order-a-lipoproteina-blood-test/> | University or government |
| Amgen Science | https://www.amgenscience.com/features/10-things-to-know-about-lipoproteina/ | Industry |
| Cleveland Clinic “LP(a) Treatment” | <https://consultqd.clevelandclinic.org/elevated-lipoproteina-is-a-long-sought-treatment-finally-on-the-way/> | University or government |
| National Lipid Association Infographic | <https://www.lipid.org/sites/default/files/files/Lp(a)%20Infographic.pdf> | Research Foundation/Non-profit |
